# An experimental study on the diagnostic advantage of dual-energy computed tomography over single-energy scan to evaluate the treatment effect following transcatheter arterial chemoembolization

**DOI:** 10.1371/journal.pone.0313543

**Published:** 2024-11-12

**Authors:** Aya Yamane, Daisuke Yasui, Hiroshi Itoh, Masayuki Kobayashi, Shin-Ichiro Kumita

**Affiliations:** 1 Department of Radiology, Nippon Medical School Musashi Kosugi Hospital, Kawasaki-shi, Kanagawa, Japan; 2 Medical Ark Inc., Koganei-shi, Tokyo, Japan; 3 Advanced Animal Medical Center, Suntou-gun, Shizuoka, Japan; 4 Department of Radiology, Nippon Medical School, Bunkyo-ku, Tokyo, Japan; University Magna Graecia of Catanzaro, ITALY

## Abstract

**Objectives:**

We assessed the diagnostic advantage of dual-energy computed tomography (DECT) over single-energy computed tomography (SECT) to evaluate lipiodol accumulation in target lesions following transcatheter arterial chemoembolization (TACE).

**Methods:**

TACE was performed in 10 rabbits in whom the VX2 tumor was implanted in their left liver lobes. The miriplatin-lipiodol mixture was injected into the common hepatic artery. All rabbits were sacrificed 2 days after TACE, and the liver was harvested. CT was performed using both single-energy and dual-energy scan modes. The specimen was stained with Oil Red O to evaluate lipiodol accumulation; this was considered the reference standard. Mutual information (MI) was used to evaluate the significance of radiological-pathological correlation. Estimated iodine content values on iodine material density images were compared with actual values obtained using mass spectroscopy.

**Results:**

Mean MI values were 0.69, 0.32, 0.83, 0.72, 0.65, and 0.58 for single-energy scan; iodine density images; and virtual monoenergetic images for energy levels of 40, 60, 80, and 100 keV, respectively. The MI value of the monochromatic image (40 keV) was the highest among all sequences. However, this was not significant compared with the single-energy scan (p = 0.81). A significant correlation was observed between the estimated and actual values of iodine content (Pearson’s product moment coefficient = 0.70, p = 0.023).

**Conclusion:**

More accurate and quantitative lipiodol evaluation in targeted tumors after TACE can be achieved by applying DECT rather than SECT.

## Introduction

According to the Barcelona Clinic Liver Cancer treatment protocol, transcatheter arterial chemoembolization (TACE) is the standard of care for inoperable hepatocellular carcinoma (HCC) [1]. A mixture of lipiodol and anticancer drugs such as doxorubicin, epirubicin, cisplatin, miriplatin, and mitomycin C is transarterially administered in conventional TACE [2, 3]. Treatment response is typically assessed based on the modified RECIST criteria, which define enhancement as viability, using contrast-enhanced computed tomography (CT) or magnetic resonance imaging (MRI) [4, 5]. CT is more commonly used in clinical practice owing to its more extensive reach than MRI. Objective response has been revealed to be a good predictor for overall survival [5]; however, enhancement is sometimes challenging to detect because of beam-hardening artifacts caused by lipiodol, leading to misdiagnosis [6]. Moreover, contrast media cannot be frequently used in patients with chronic renal failure, especially in the elderly population.

Considering that dense lipiodol accumulation reflects necrosis, evaluating lipiodol accumulation in the tumor via plain CT can be a surrogate marker [7]. The Hounsfield unit (HU) has been measured using a single-energy scan; however, dual-energy CT-based virtual monoenergetic images for the low-energy band can be used to improve the contrast between the liver parenchyma and lipiodol [8, 9]. Moreover, quantitatively evaluating lipiodol accumulation can be performed using material decomposition, which may be more precise than the conventional qualitative evaluation [9]. However, only a limited number of articles have referred to the usefulness of dual-energy CT in evaluating tumor response to TACE. Furthermore, estimating the amount of lipiodol accumulated in the tumor tissue using iodine material density images has not been verified.

Thus, this study aimed to reveal the usefulness of virtual monoenergetic images and iodine density maps to qualitatively and quantitatively evaluate lipiodol accumulation in tumors following TACE.

## Materials and methods

The following study protocol was prepared prior to the experiment; however, it has not been registered.

### TACE

The study was performed in strict accordance with local acts and guidelines, including the Welfare and Management of Animals Act. The study protocol was approved by the Animal Experiment Ethics Committee of the Nippon Medical School (Tokyo, Japan; protocol number: 30–014). Maximum efforts were made to reduce suffering during the procedure. VX2 tumor-bearing Japanese white rabbits (14 weeks old, female, clean; Japan SLC, Inc., Hamamatsu, Japan) were used. The VX2 tumor is an anaplastic squamous cell carcinoma induced by infection with the Shope papilloma virus in rabbits and is commonly used as an experimental liver cancer model. The tumor was extracted 4 weeks after implantation in the posterior leg of Japanese white rabbits. The purified tumors were homogenized by adding 20 mL of normal saline. Five-fold-diluted cell suspension obtained after centrifugation (approximately 2–3 min, 500 rpm) was injected into the left liver lobe of each rabbit. The entire procedure, from culture to tumor implantation, was performed at Japan SLC, and tumor-bearing rabbits were supplied to our institute. TACE was performed 14 days after tumor implantation in 10 rabbits.

Veterinary physicians took care of all rabbits before, during, and after the procedure. Angiographic procedures were performed under general anesthesia, which was induced using a subcutaneous medetomidine hydrochloride (0.1 mg/kg) and ketamine hydrochloride (25 mg/kg) injection and was maintained via inhalation of 2%-2.5% isoflurane. Each rabbit was monitored using a pulse oximeter and electrocardiography. A 4-F introducer sheath (Medikit Co., Ltd., Tokyo, Japan) was inserted into the surgically exposed common femoral artery. The celiac artery was cannulated using a 4-F C2 diagnostic catheter (Medikit Co., Ltd.), and subsequently, a 2-F microcatheter (Gold Crest Neo; HI-LEX, Takarazuka, Japan) was coaxially advanced into the left hepatic artery. Miriplatin (Dainippon Sumitomo Pharma, Osaka, Japan) and lipiodol (Guerbet, Aulnay-sous-Bois, France) mixture (0.1 mL) were injected under fluoroscopic guidance without subsequent embolization.

All rabbits were individually housed in animal cages and maintained under appropriate temperature and lighting control: 12-hour light and dark cycles. Free access to standard laboratory food and water was guaranteed, and rabbits were closely monitored once every 3 hours and checked for unintended adverse events. Meloxicam (Metacam) was subcutaneously injected at a dose of 0.2 mg/kg body weight for analgesia when rabbits exhibited signs of discomfort or were pyretic. The rabbit was supposed to be euthanized within 6 hours if it showed any sign of impending death: poor feeding, continuous crouching, forced breathing, continuous vomiting, severe diarrhea, and continuous gasping. The rabbits were otherwise sacrificed 2 days after TACE via an intravenous injection of pentobarbital sodium (100 mg/kg), and the liver was harvested.

### CT

CT of the extracted liver was performed using Revolution HD (GE Healthcare, Waukesha, WI, USA). The tumor was extracted from the liver and dissected into two parts: one for iodine content measurement using mass spectroscopy and the other for pathological evaluation. Single- and dual-energy scans were performed using the following parameters: 400 mA tube current, 120 kVp tube voltage, 1 second rotation speed, 0.625 mm slice thickness, 0.531 helical pitch, 12 cm FOV, and reconstruction Standard Plus with no iterative reconstruction for single-energy scan; 600 mA tube current and 80/140 kVp tube voltage with the same parameters for dual-energy scan. Iodine material density and virtual monochromatic images at 40, 60, 80, and 100 keV were synthesized.

### Mass spectroscopy

Iodine concentration was evaluated using inductively coupled plasma mass spectroscopy (Japan Testing Laboratories, Inc., Ogaki, Japan).

### Pathological evaluation

Lipid staining using Oil Red O was performed to quantitatively evaluate lipiodol accumulation in the tumors. The maximum tumor cross-section was used for evaluation. Specimens were digitized using whole-slide imaging (NanoZoomer XR; Hamamatsu Photonics K.K., Yokohama, Japan). The scanned images were compressed to obtain a JPEG image with a resolution of 1920 × 984 pixels. Lipiodol accumulation was evaluated using the HSV model, which defines the color space using three parameters: hue, saturation, and value. The lesions without lipids were bluish. An area with a “hue” value > 55 and < 65 was digitally subtracted from the digitized specimen by setting the value to zero. The remaining area was defined as the region of lipiodol accumulation. The lipiodol accumulation extent was evaluated using “saturation.” The square region of interest was defined on CT and pathological specimen images, which corresponded to the area of the target lesion. Pixel sizes of pathological specimen images were larger than those of CT images. Thus, pathological specimen images were resized to fit the pixel size of CT images using the interpolation function of OpenCV 4 (cv2.INTER_AREA). The source codes were written in Python (Anaconda 4.6.14; Anaconda, Inc., Austin, TX, USA) using a Jupyter Notebook, whereas the cv2 module was used for image processing, as previously described.

### Radiopathological correlation

The HU values for CT and saturation values for pathological specimens were scaled to 256 grades. Mutual information (MI), defined using a formula subsequently mentioned, was used to evaluate the similarity between the scaled HU and saturation values. The proportion of pixels that had a specific value on CT and pathological specimen images to the total pixel number was defined as p(x, y), whereas the proportion of pixels that had a specific value on either CT or pathological specimen images to the total pixel number was defined as p(x) and p(y), respectively. The correlation between the iodine content measured using mass spectroscopy (concentration multiplied by the mass of the specimen) and that estimated using the iodine material density images (sum of each pixel value in the region of interest) was compared.


$$I(X;Y)=\sum_{y\in Y}\sum_{x\in X}p(x,y)log\frac{p(x,y)}{p(x)p(y)}$$

### Statistical analysis

A one-way analysis of variance was performed to compare the MI between the iodine material density, single-energy scan, and virtual monochromatic images with different keV (40, 60, 80, and 100). A post-hoc analysis using Tukey’s test was subsequently performed. Pearson’s product-moment correlation was used to evaluate the correlation between the iodine content measured using mass spectroscopy and that estimated using the iodine material density images. Statistical significance was set at p < 0.05. All analyses were performed using the Python scipy.stats module (version 1.11.2).

## Results

**Fig 1:**
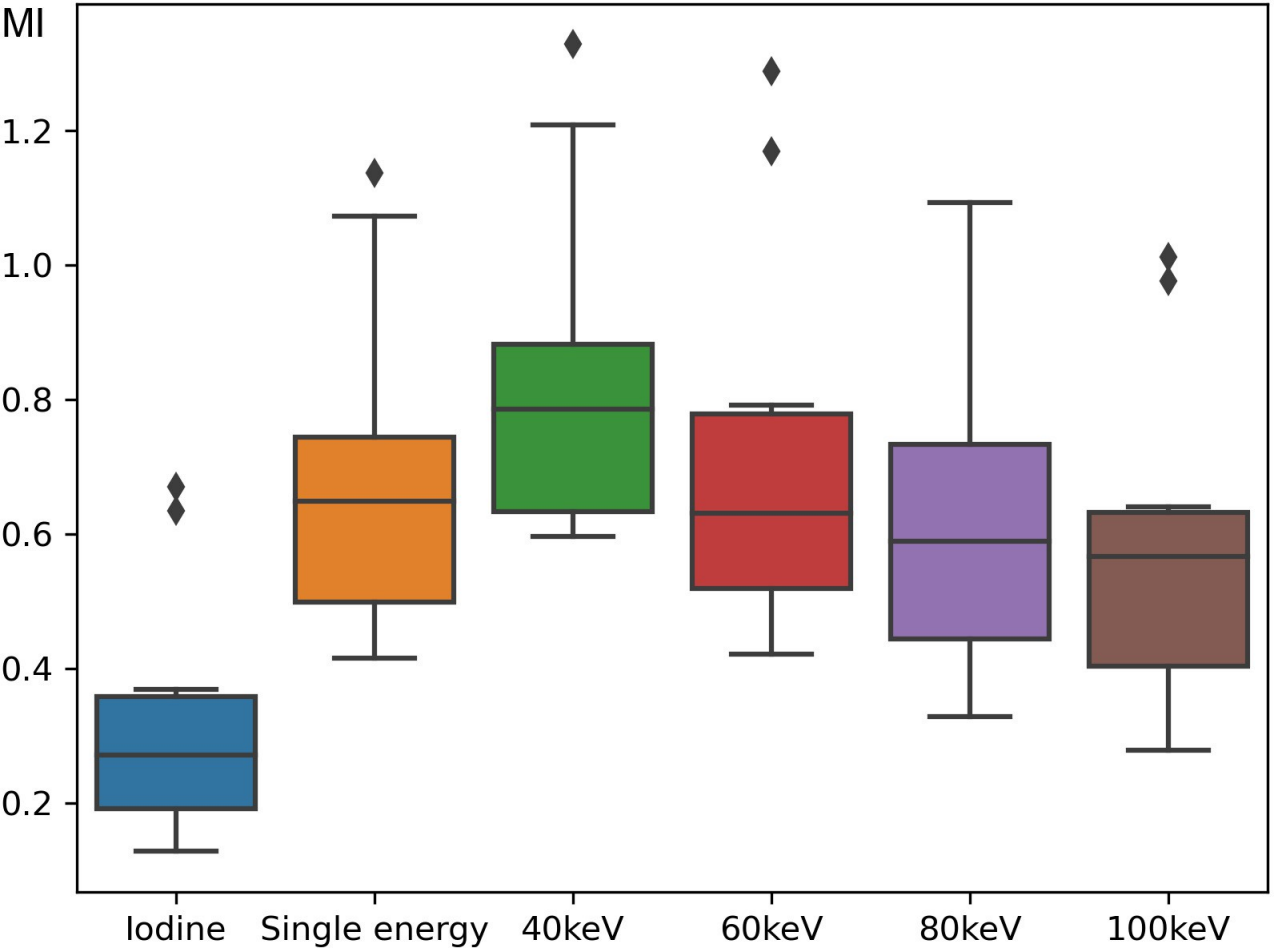
Mutual information of each protocol. Iodine material density image; single-energy scan; and virtual monochromatic image at 40, 60, 80, and 100 keV are illustrated. MI, mutual information.

**Fig 2:**
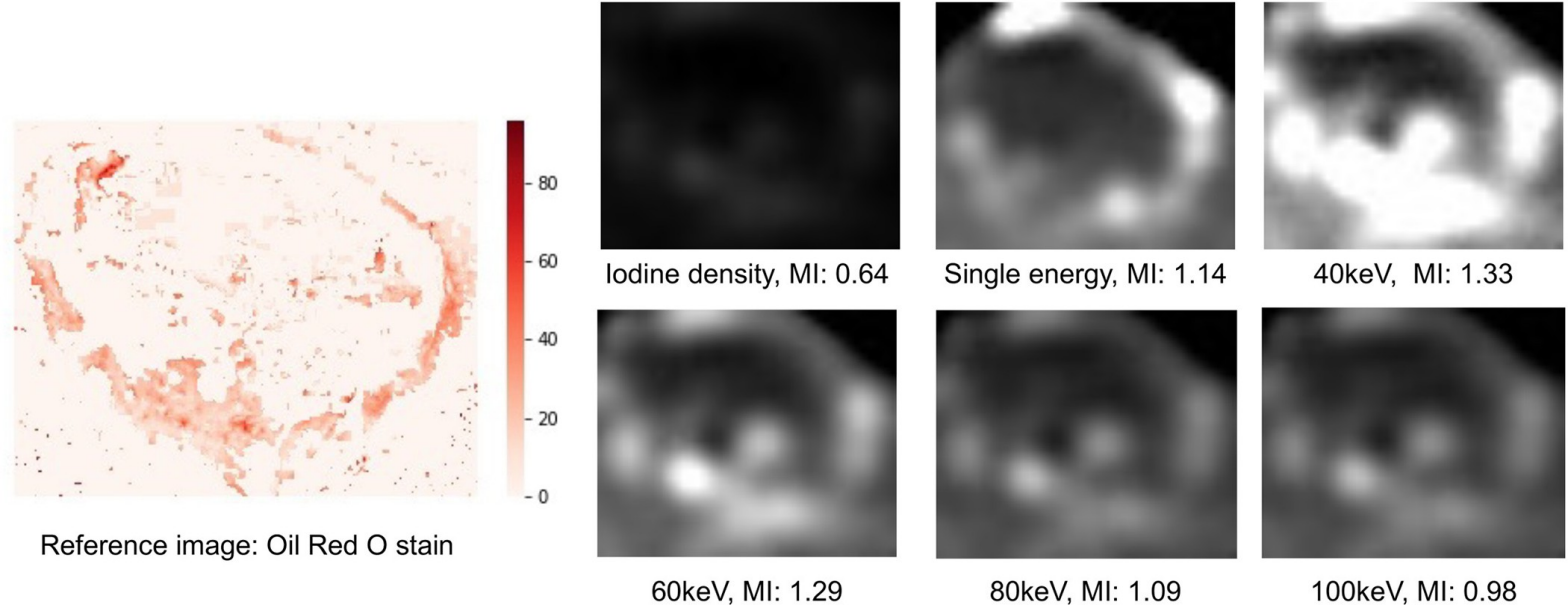
An example of radiology-pathology correlation of lipiodol accumulation. Oil Red O stained images were used as reference, and computed tomography images from various protocols were compared for a representative specimen. MI, mutual information.

**Fig 3:**
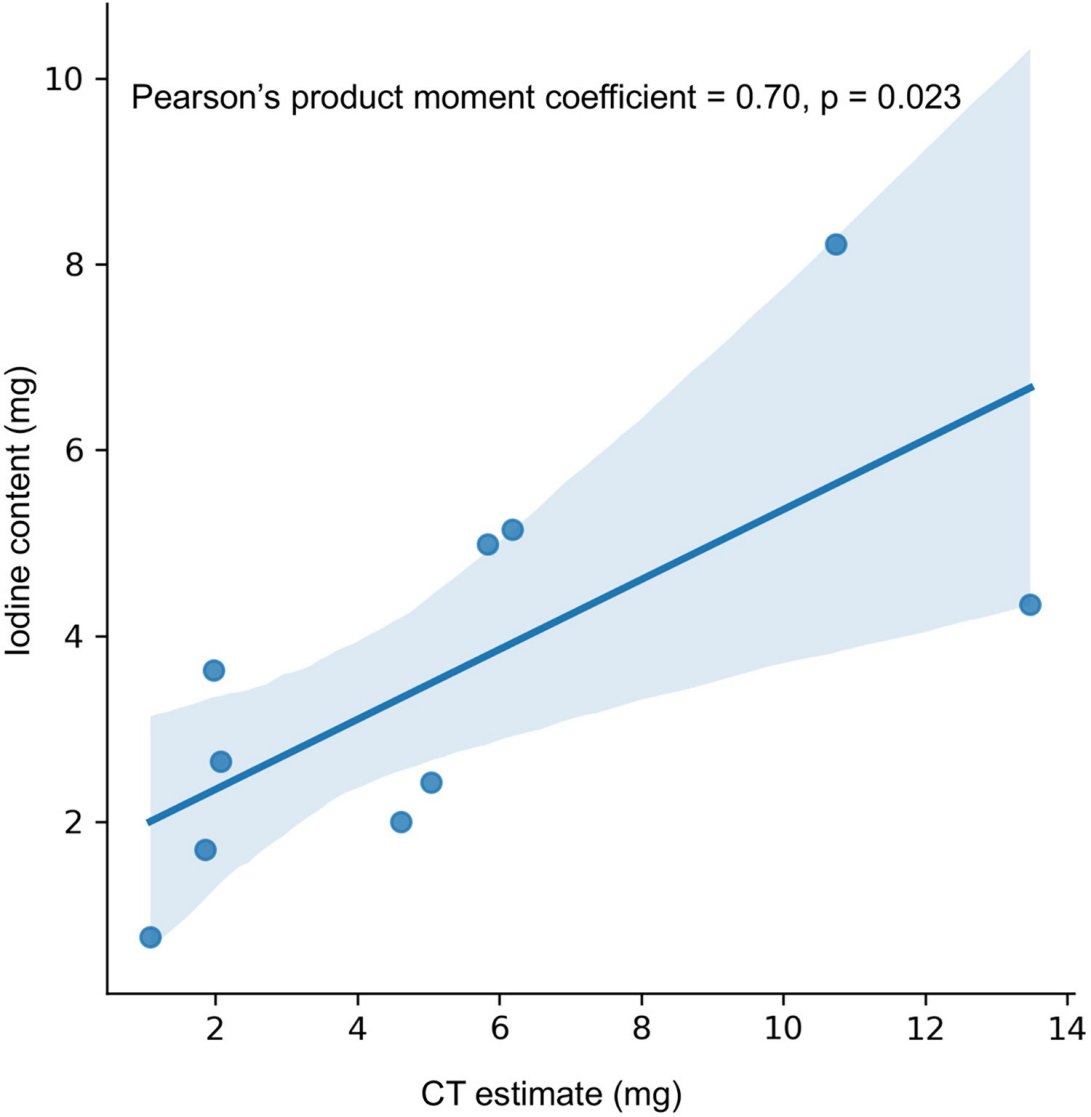
Correlation of the measured iodine content and estimated value from computed tomography images. A scatter plot of the iodine content measured using mass spectroscopy and the estimated values from the iodine material density images are illustrated. The blue line indicates the linear regression model, and the blue area represents the 95% confidence interval.

No animal or specimen was excluded from the experiment or evaluation before, during, or after the experiment. All animals survived the procedure and were evaluated. Mean MI values were 0.69, 0.32, 0.83, 0.72, 0.65, and 0.58 for single-energy scan; iodine material density images; and virtual monoenergetic images for each energy level: 40, 60, 80, and 100 keV, respectively (Fig 1). Significant differences were observed in MI values among all sequences (p = 0.00099). However, post hoc analysis revealed a significant difference between the iodine material density images and single-energy scan (p = 0.019), iodine material density images and 40 keV images (p = 0.00040), and iodine material density images and 60 keV images (p = 0.0087), whereas the difference between the other two groups was insignificant. The MI value was the highest in the 40 keV images; however, the difference was insignificant compared with the single-energy scan (p = 0.81, Fig 2). A significant correlation was observed between the estimated and actual values of the iodine content (Pearson’s product moment coefficient = 0.70, p = 0.023, Fig 3).

## Discussion

Transcatheter arterial embolization is considered the standard of care for inoperable HCC. It can also be applied for cancer-related hemorrhage with a high clinical success rate [10]. Modified RECIST criteria, commonly used to assess local tumor control after TACE, may not be applicable to patients with renal dysfunction owing to their reliance on intravenous contrast media administration. Moreover, these criteria overlook the accumulation of anticancer agents/lipiodol in the target tumor. A more sophisticated embolization agent, a mono-disperse emulsion, has shown promise, compared with conventional emulsions with initially promising results [11, 12]. This homogenous emulsion was composed of an anticancer agent solution and lipiodol, obtained by pushing the solution into Lipiodol through a hydrophilic SPG membrane with even-sized pores. Compared with conventional emulsion obtained by manual mixing of the anticancer solution and lipiodol using a three-way stopcock, the mono-disperse emulsion has demonstrated improved accumulation in the tumor. Accurately evaluating lipiodol accumulation in the targeted lesion is now crucial.

Conventional single-energy CT measures HU values in the targeted lesions but lacks accuracy owing to the nature of multichromatic X-rays. HU values reflect the linear attenuation coefficient; thus, they depend on the energy of the irradiated X-rays. X-rays reaching the targeted tumor tend to have a high energy level (beam hardening) because X-rays with low energy levels tend to be absorbed by the tissue before entering the liver. Variations in the energy spectrum of X-rays reaching the targeted lesion despite fixed tube current and voltage peak settings render the estimation of lipiodol accumulation inaccurate when relying on HU values. Additionally, streaking artifacts caused by lipiodol may impact HU values. While virtual monochromatic images and estimating lipiodol in tumors using iodine material imaging with dual-energy CT can offer potential solutions, validation through additional studies is required [13].

This study demonstrated a strong correlation between HU values from virtual monochromatic images at low energy levels (40 and 60 keV) and histological lipiodol accumulation. Additionally, estimated lipiodol content from iodine material density images correlated well with mass spectroscopy measurements. These findings suggest that qualitative assessment with low-energy virtual monochromatic images and quantitative evaluation using iodine material density images are suitable for assessing lipiodol accumulation after TACE, consistent with the results of some previous studies. However, some limitations need consideration.

First, the suitability of saturation values in HSV models as the reference standard for assessing radiopathological correlations remains uncertain. Quantitative evaluations in this regard are scarce, hindering the establishment of a standard method. While this method appears plausible owing to the relatively high MI of a single energy scan (0.69), its validation requires additional research.

Second, the tube current employed in this study exceeded the levels that can be used in clinical practice, raising concerns about radiation exposure. Additionally, beam-hardening effects were overlooked. Given X-ray attenuation in the human body, a risk of absorption may exist before reaching the liver at 40 keV, potentially elevating noise. Thus, determining the ideal X-ray energy level necessitates further clinical investigation.

Third, photon-counting detector (PCD) CT could offer better suitability for assessing lipiodol accumulation owing to its ability to directly count photons at specific energy levels reaching the detector [14]. Therefore, the interpretation of this study’s findings may evolve as PCD becomes more widespread in the future.

In conclusion, virtual monochromatic images with low energy levels and iodine material density images using dual-energy CT prove valuable in the qualitative and quantitative assessment of lipiodol after TACE for HCC; however, additional research is necessary.

## Supporting information

S1 Data Raw data related to Fig 1 are presented. (XLSX)

S2 Data Raw data related to Fig 3 are presented. (XLSX)
